# Integrative Analysis Reveals the Potential Role and Prognostic Value of GOLM1 in Hepatocellular Carcinoma

**DOI:** 10.1155/2022/8284500

**Published:** 2022-09-28

**Authors:** Yan Lin, Ziqin He, Xing Gao, Lu Lu, Cheng Lu, Julu Huang, Min Luo, Jiazhou Ye, Rong Liang

**Affiliations:** ^1^Department of Digestive Oncology, Guangxi Medical University Cancer Hospital, Nanning, Guangxi 530021, China; ^2^Department of Hepatobiliary Surgery, Guangxi Medical University Cancer Hospital, Nanning, Guangxi 530021, China

## Abstract

Overexpression of Golgi membrane protein 1 (GOLM1) is closely associated with hepatocellular carcinoma (HCC) vascular invasion. How GOLM1 may be involved in angiogenesis in HCC remains unclear. We explored how GOLM1 promotes angiogenesis in HCC and potential prognostic value. Expression levels of GOLM1 in HCC patients and healthy controls were obtained from The Cancer Genome Atlas (TCGA). Differentially expressed genes (DEGs) between HCC patients and controls were compared. GOLM1 was knocked out in the HCC cell line, and RNA sequencing and DEG expression analysis were performed compared with control cells. Based on TCGA data and cell line RNA sequencing data, DEGs affected by a high expression of GOLM1 were identified. Subsequently, enrichment analysis was performed to explore the functions and pathways of the DEGs affected by a high expression of GOLM1. A relevant network analysis was built. Cox regression, genomic variance analysis scores, minimum absolute shrinkage and selection operator regression, and random forest regression models were applied to determine the best prognostic model and validated using the GSE54236 dataset from the Gene Expression Omnibus (GEO). We determined the effect of GOLM1 expression on immune cell infiltration in liver cancer. GOLM1 was overexpressed in HCC tissues compared with controls, and its level correlated with tumor purity and prognosis. 400 DEGs affected by highly expressed GOLM1 were identified in TCGA and cell line RNA sequencing data. Enrichment analysis revealed that these DEGs may be related to biological processes of oxidative stress and angiogenesis and involved in the VEGF signaling pathway and protein processing in endoplasmic reticulum. We predicted a comprehensive regulatory network in which GOLM1 activated VEGF signaling to promote HCC angiogenesis. GOLM1 may interact with E2F1 and IGF2BP3 to promote angiogenesis. GOLM1 overexpression was associated with greater immune cell infiltration. A random forest regression model was the best prognostic model. Our study reveals a potential molecular mechanism of GOLM1 in promoting HCC. We developed two prognostic models based on DEG associated with GOLM1 overexpression to help stratify HCC prognosis and improve individualized treatment.

## 1. Introduction

Liver cancer has the fourth highest mortality among malignant tumors worldwide [1]. Among the three pathological types of primary liver cancer, hepatocellular carcinoma (HCC) is the most common, accounting for about 90% of cases [2]. HCC is a typical vascular tumor, so angiogenesis plays an important role in its onset and progression [3]. Several angiogenesis pathways are abnormally activated in HCC to support tumor development, including pathways involving vascular endothelial growth factor (VEGF), fibroblast growth factor (FGF), and platelet-derived growth factor and their receptors, as well as pathways involving angiopoetin and Tie [4]. Targeted antiangiogenesis therapy has become one of the main strategies for treating HCC. Although a variety of antiangiogenic drugs are currently under development, only sorafenib and lenvatinib have been approved for the first-line treatment of advanced HCC [2–4]. Therefore, in-depth studies on the mechanisms of HCC angiogenesis are needed to identify new targets for the development of effective antiangiogenic drugs.

We wondered whether Golgi membrane protein 1 (GOLM1), also known as GP73 or GOLPH2, may be a suitable therapeutic target in HCC. GOLM1 is expressed predominantly in epithelial cells [5] and can also be cleaved by proprotein convertase and secreted into the blood [6]. GOLM1 is overexpressed in a variety of malignancies including HCC, and its high expression correlates strongly with poor prognosis [7]. A previous multicenter study comparing serum GOLM1 and alpha-fetoprotein (AFP) in 4217 human subjects showed that GOLM1 had a sensitivity and specificity of 76.4% and 97.4%, respectively, for HCC, while AFP had a sensitivity and specificity of 58.2% and 85.3%, respectively [8]. Another meta-analysis involving 11 studies showed that GOLM1 was superior to AFP as a diagnostic marker in 5 studies, while the results were opposite or unclear in the remaining 6 studies [9]. Therefore, GOLM1 may even allow a more sensitive and specific diagnosis of liver cancer than AFP [8–10].

Our previous study found that serum GOLM1 levels were significantly higher in patients with HCC, and its sensitivity and specificity for diagnosing HCC were higher than those of AFP [11]. Further studies related to GOLM1 and drug resistance in HCC were conducted, and it was confirmed that GOLM1 promoted oxaliplatin resistance in human HCC cells [12]. In addition, it was also noted during the collection of clinical case data from HCC patients that GOLM1 elevated vascular invasion more significantly in HCC patients [13]. Other studies reported that GOLM1 promotes tumor metastasis by participating in the epithelial-mesenchymal transformation and recycling of epidermal growth factor receptor and receptor tyrosine kinases [14, 15]. Moreover, GOLM1 can enhance STAT3 phosphorylation by upregulating the epidermal growth factor receptor and then activating programmed death-ligand 1 transcriptional expression to inhibit immune responses [16]. Thus, evidence suggests that GOLM1 promotes the pathogenesis and progression of HCC through various mechanisms, whereas no detailed studies specifically addressing the GOLM1 gene and HCC angiogenesis have been reported both nationally and internationally.

To explore this possibility, we combined data from The Cancer Genome Atlas-Liver Hepatocellular Carcinoma (TCGA-LIHC) and experimental sequencing data from an HCC cell line. These findings may reveal new targets and strategies for targeted antiangiogenic therapy in HCC.

## 2. Material and Methods

### 2.1. Data Collection

Gene expression profiles were obtained from publicly available databases: The Cancer Genome Atlas (TCGA; https://portal.gdc.cancer.gov), from which data on 371 HCC patients and 50 controls were extracted and Gene Expression Omnibus (GEO; http://www.ncbi.nlm.nih.gov/geo), from which the dataset GSE54236 [17] was extracted for 81 tumor tissues from 78 HCC patients and 80 controls from 54 consecutive patients with cirrhosis. GEO data were used as the validation dataset. The current study adheres to TCGA and GEO data access policies and publication guidelines. The Tumor Immune Estimation Resource database (TIMER, http://timer.cistrome.org/) was to identify the differential expression of GOLM1 in tumor and normal tissues of multiple cancer species.

### 2.2. Immunohistochemistry (IHC)

Tumor and adjacent tissues from three patients with HCC were collected from the Guangxi Medical University Cancer Hospital, immediately fixed in 10% formaldehyde for 12 h, dehydrated, transparent, paraffin-embedded, and sectioned (4 *μ*m) for IHC. After dewaxing the paraffin sections into water, antigen repair was performed using sodium citrate buffer (pH 6.0) for 15 min at 95°C and washed 3 times with phosphate buffered saline (PBS). The slides were then incubated with 3% H_2_O_2_ for 30 min to block endogenous peroxidase activity and 5% bovine serum albumin (BSA) for 1 h at room temperature to block nonspecific binding sites and incubated with primary antibody GOLM1 (American, Proteintech, Cat No. 15126-1-AP) overnight at 4°C. After three washes with PBS, the sections were incubated with the corresponding horseradish peroxidase at 37°C. The sections were incubated with the corresponding horseradish peroxidase- (HRP-) coupled secondary antibody (China, Beijing, ZSGB-BIO, SP-9001) in a wet box for 1 h and washed three times with PBS. Diaminobenzidine (DAB) was incubated for 10 min for color development, and hematoxylin was incubated for 3 min for nuclear restaining. Finally, the gradient was dehydrated in ethanol, clear in xylene, and sealed in neutral gum. IHC images were acquired under a standard light microscope (Olympus, Tokyo, Japan) and analyzed using Image-Pro Plus software (Media Cybernetics, Rockville, MD, United States). This study was approved by the Ethics Committee of the Guangxi Medical University Cancer Hospital. All procedures involving human participants complied with the ethical standards of the research committee and its ethical standards. Informed consent was obtained from participants for all study procedures and sequencing protocols.

### 2.3. Cell Cultures

We purchased MHCC97H cells from the Shanghai Institutes for Biological Sciences of the Chinese Academy of Sciences (CAS) as an HCC in vitro model. After being thawed, resuscitated, and passaged, the cells were kept in RPMI 1640 medium containing 10% fetal bovine serum and cultured in 37°C, 5% CO_2_ saturated humidity cell incubator.

### 2.4. RNA Sequencing

GOLM1 expression was silenced in MHCC97H cells using two small interfering RNAs (siRNAs). The siRNA sequences were: siRNA 1, 5′-agggaaacgtgcttggtaa-3′ and siRNA 2, 5′-gaatagaagaggtcaccaa-3′. Lentiviral vectors encoding short hairpin RNA (shRNA) were designed based on the siRNA sequences to knock down GOLM1 expression (GOLM1-KD) [12]. These vectors were constructed by Hanyin Co. (Shanghai, China). The lentiviruses expressing the negative control lentivirus (Vector) were also constructed by Hanyin Co. (Shanghai, China). Total RNA was isolated from GOLM1-KD MHCC97H cells and control MHCC97H cells using TRIzol (Thermo Scientific, Uppsala, Sweden) and purified using the RNeasy kit (Qiagen, Valencia, CA). RNA-Seq libraries were constructed using the TruSeq Stranded mRNA-Seq Library Preparation Kit (Illumina, San Diego, California, USA). Samples were sequenced using the Illumina NovaSeq system, generating paired-end reads of 150 base pairs. Raw sequence reads were converted into fragments per exon kilobase per million mapped reads (FPKM) to quantify gene expression.

### 2.5. Survival Analysis

Expression profiles were normalized using the “voom” function in the limma package in R [18]. For analyzing survival as a function of GOLM1 expression, the best cut-off value was determined using the “surv_cutpoint” function in the survminer package in R (http://www.rstudio.org). According to the best cut-off value, patients were divided into groups expressing low or high levels of GOLM1. Then, overall survival (OS) was compared between the two groups using the log rank test.

### 2.6. Identification of Differentially Expressed Genes (DEGs)

The limma package in R was used to identify DEGs between HCC patients and controls in TCGA data, as well as mRNAs whose expression differed between GOLM1-KD and control cells. DEGs with expression differences showing *P*.adjust < 0.05 were considered significant and included in further analyses. DEGs that were up- or downregulated across both TCGA and RNA sequencing data were defined as DEGs affected by GOLM1-KD. DEGs whose expression was opposite to that of DEGs affected by GOLM1-KD were identified as genes associated with GOLM1 overexpression.

### 2.7. Functional Enrichment Analysis

To explore the biological processes and signaling pathways in which DEG associated with GOLM1 overexpression may be involved, Gene Ontology (GO) and Kyoto Encyclopedia of Genes and Genomes (KEGG) analyses were performed using the clusterProfiler package in R [19]. Results associated with *P* < 0.05 were defined as significant.

### 2.8. Constructing Regulatory Networks

We used the RNAInter database (http://www.rna-society.org/rnainter) [20] to extract DEGs that interacted with GOLM1 (*P* < 0.05). The set screening criterion was a score > 0.5. In combination with KEGG pathway genes, the Pearson correlation test and the hypergeometric test were utilized using the expression profiles. Finally, a comprehensive regulatory network of GOLM1-KEGG correlation was obtained. Results associated with *P* < 0.05 were considered significant.

### 2.9. Molecular Docking

We then explored whether target proteins may be able to bind GOLM1. We downloaded the three-dimensional structures of GOLM1 and target proteins from the Protein Data Bank (http://www.rcsb.org). Molecular docking was performed using Hex 8.0.0 software [21], and the results were visualized with PyMol software [22]. For whether two molecules have binding ability between them, when the docking energy is less than 0 KJ/mol, it means that both have binding potential, and the smaller the energy, the higher the binding potential.

### 2.10. Gene Set Variant Analysis (GSVA)

We extracted the mechanism genes for univariate Cox regression analysis to obtain the meaningful DEGs of univariate Cox regression and then calculated the GSVA score of these genes for individual sample using GSVA package in R [23].

### 2.11. Least Absolute Shrinkage and Selection Operator (LASSO) Regression Models

Cox regression was used to identify DEG associated with OS of HCC patients. The glmnet package in R [24] was used to integrate potentially prognostic DEGs into a binomial LASSO regression model. During LASSO regression, we retained potential predictors with nonzero coefficients in order to generate candidate DEGs. Areas under receiver operating characteristic curves (AUCs) were calculated using the pROC package in R [25]. Cox regression was used to construct a prognostic nomogram to predict two- and five-year OS of HCC patients in TCGA.

### 2.12. Random Forest Algorithm

Genes with prognostic value in the univariate Cox regression were obtained. Survival data were dimensionally reduced using a random forest survival algorithm [26], ranked based on factor importance, and then filtered for gene signatures. Forest plots were generated using the forestplot package in R.

### 2.13. Immune Cell Infiltration

The level of infiltration of different types of immune cells was assessed using CIBERSORT (https://cibersort.stanford.edu/) and ssGSEA in the GSVA routine. Immune cells indicated as 0 were excluded from the analysis. A set of marker genes for the immune cell types analyzed by ssGSEA was obtained from Bindea et al. [27]. TIMER 2.0 [28] was used to assess the levels of immune cell infiltration. We also evaluated potential correlations between candidate genes and immune cell types using Pearson correlation analysis, with significance defined as *P* < 0.05. |cor| > 0.2 was considered to indicate that a correlation existed.

### 2.14. Data Analysis and Statistics

All bioinformatics analyses in this study were performed based on the Bioinforcloud platform (http://www.bioinforcloud.org.cn).

## 3. Results

### 3.1. GOLM1 Is Overexpressed in HCC and Strongly Associated with Poor Patient Prognosis

**Figure 1:**
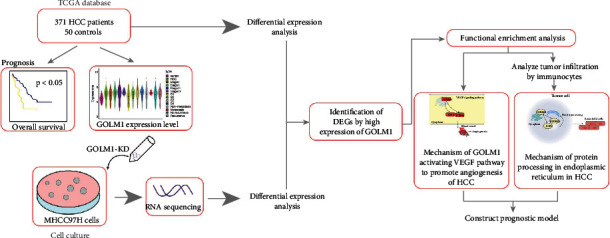
Study flowchart. The flow diagram of this study. DEGs: differentially expressed genes; HCC: hepatocellular carcinoma; KD: knocked down; TCGA: The Cancer Genome Atlas; VEFG: vascular endothelial growth factor.

**Figure 2:**
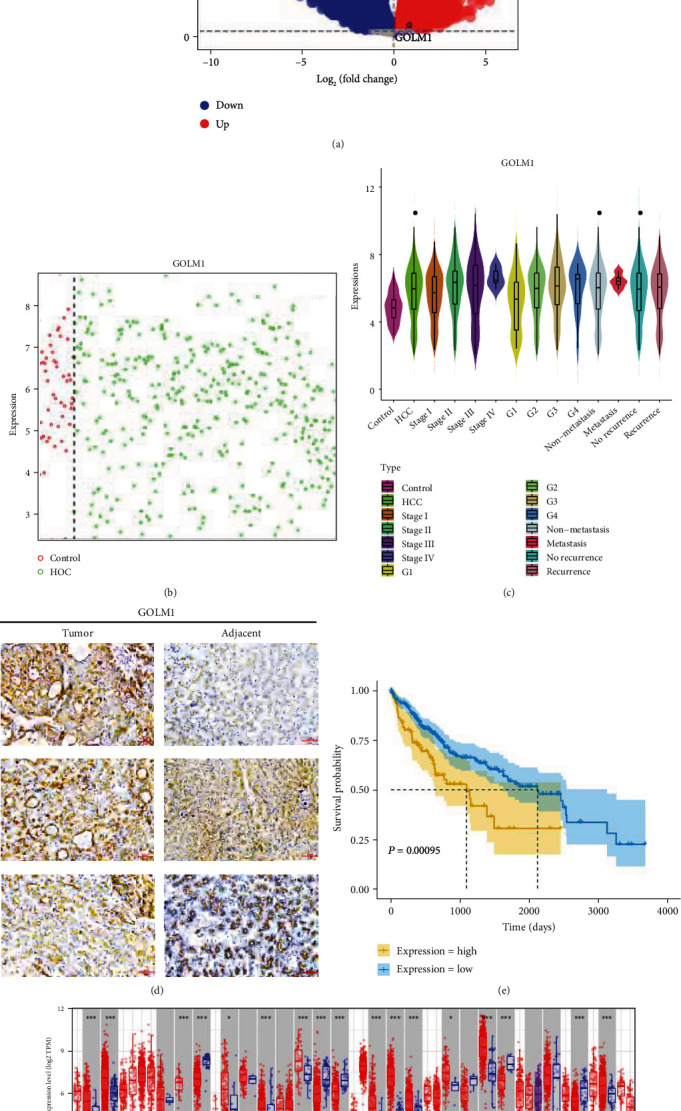
GOLM1 is overexpressed in hepatocellular carcinoma (HCC) and strongly associated with poor patient prognosis. (a) Differentially expressed genes (DEGs) between HCC patients and healthy controls from The Cancer Genome Atlas (TCGA). (b) GOLM1 expression level in 371 HCCs (green circle) and 50 adjacent normal tissues (red circle) from the TCGA. (c) GOLM1 expression at different HCC stages compare to the control group. (d) Immunohistochemistry against GOLM1 in tumor and adjacent tissues. (e) Kaplan-Meier curves of overall survival of patients in the high and low GOLM1 groups in TCGA. (f) GOLM1 expression in the Tumor Immune Estimation Resource database. DEGs: differentially expressed genes.

The study flowchart is shown in Figure 1. We identified a total of 12040 DEGs between HCC and controls in TCGA data (Figure 2(a)). In TCGA specimens (371 HCC tissues and 50 healthy controls), we found that GOLM1 was abundantly expressed at different stages of HCC but weakly expressed in controls (Figures 2(b) and 2(c)). Similarly, immunohistochemistry analysis showed a higher expression of GOLM1 in tumor than in adjacent tissue (Figure 2(d)). Interestingly, HCC patients with high expression of GOLM1 had poorer OS than those with low expression (Figure 2(e)).

Furthermore, analysis of the TIMER database showed GOLM1 to be upregulated in several types of tumors (Figure 2(f)). The above results show that GOLM1 is highly expressed in HCC. In addition, GOLM1 expression is closely related to the poor prognosis of HCC.

### 3.2. Biological Functions of DEG Associated with GOLM1 Overexpression in HCC

**Figure 3:**
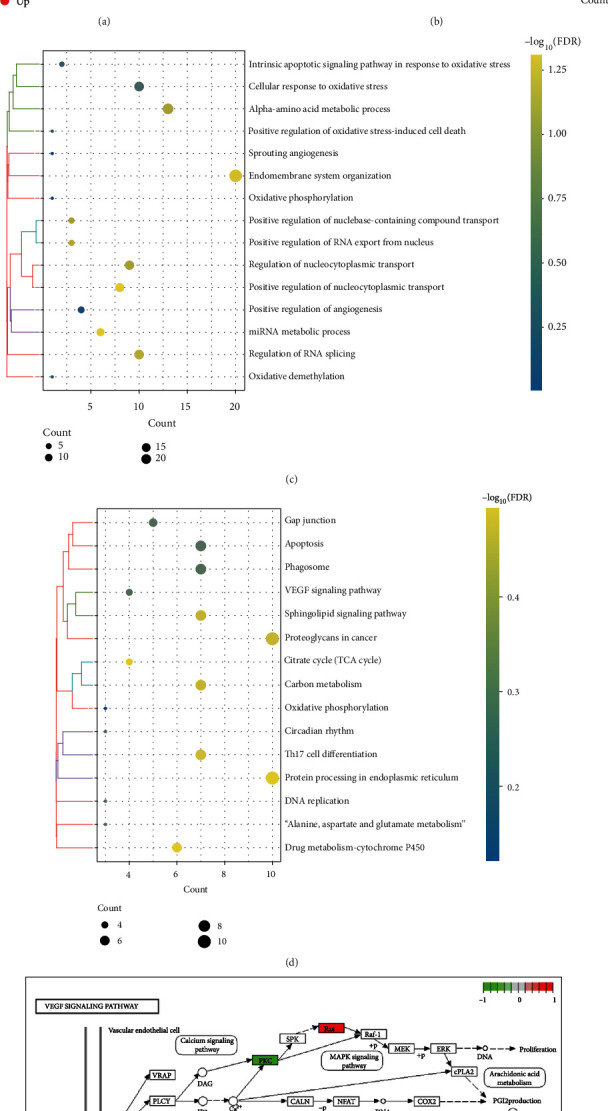
Identification and enrichment analysis of genes associated with high GOLM1 expression. (a) DEGs in GOLM1-knocked down (KD) MHCC97H cells and control MHCC97H cells. (b) DEGs between TCGA and RNA sequencing data. (c, d) GO and KEGG enrichment analyses of 400 specific DEGs associated with GOLM1 overexpression in HCC. (e) KEGG pathway annotations of the VEGF signaling pathway. Green indicates low expression, and red indicates high expression. DEGs: differentially expressed genes; GO: Gene Ontology; VEGF: vascular endothelial growth factor.

To identify DEG associated with GOLM1 overexpression, we performed differential expression analysis of the RNA sequencing data from our cell cultures. A total of 1363 DEGs between the GOLM1-KD MHCC97H cells and control MHCC97H cells groups were identified, comprising 744 upregulated and 619 downregulated genes (Figure 3(a)). Then, we analyzed overlapping DEGs found in 1363 DEGs cell cultures and the 14787 DEGs identified in TCGA. We found 737 overlapping genes, which were defined as DEGs affected by GOLM1-KD. In addition, 400 DEGs opposite to the DEGs affected by GOLM1-KD expression were identified as the specific DEGs associated with GOLM1 overexpression (Supplementary Table 1, Figure 3(b)).

These specific DEGs associated with GOLM1 overexpression were enriched for GO biological processes related to oxidative stress and angiogenesis: cellular response to oxidative stress, intrinsic apoptotic signaling pathway in response to oxidative stress, oxidative phosphorylation, and positive regulation of angiogenesis (Figure 3(c)). KEGG pathway analysis showed that these DEGs were involved mostly in protein processing in the endoplasmic reticulum, oxidative phosphorylation, apoptosis, and the VEGF signaling pathway (Figure 3(d)). Among them, the VEGF signaling pathway is activated in HCC and promotes angiogenesis [29], which attracted our attention (Figure 3(e)).

### 3.3. GOLM1 Activates the VEGF Signaling Pathway to Promote Angiogenesis in HCC

**Figure 4:**
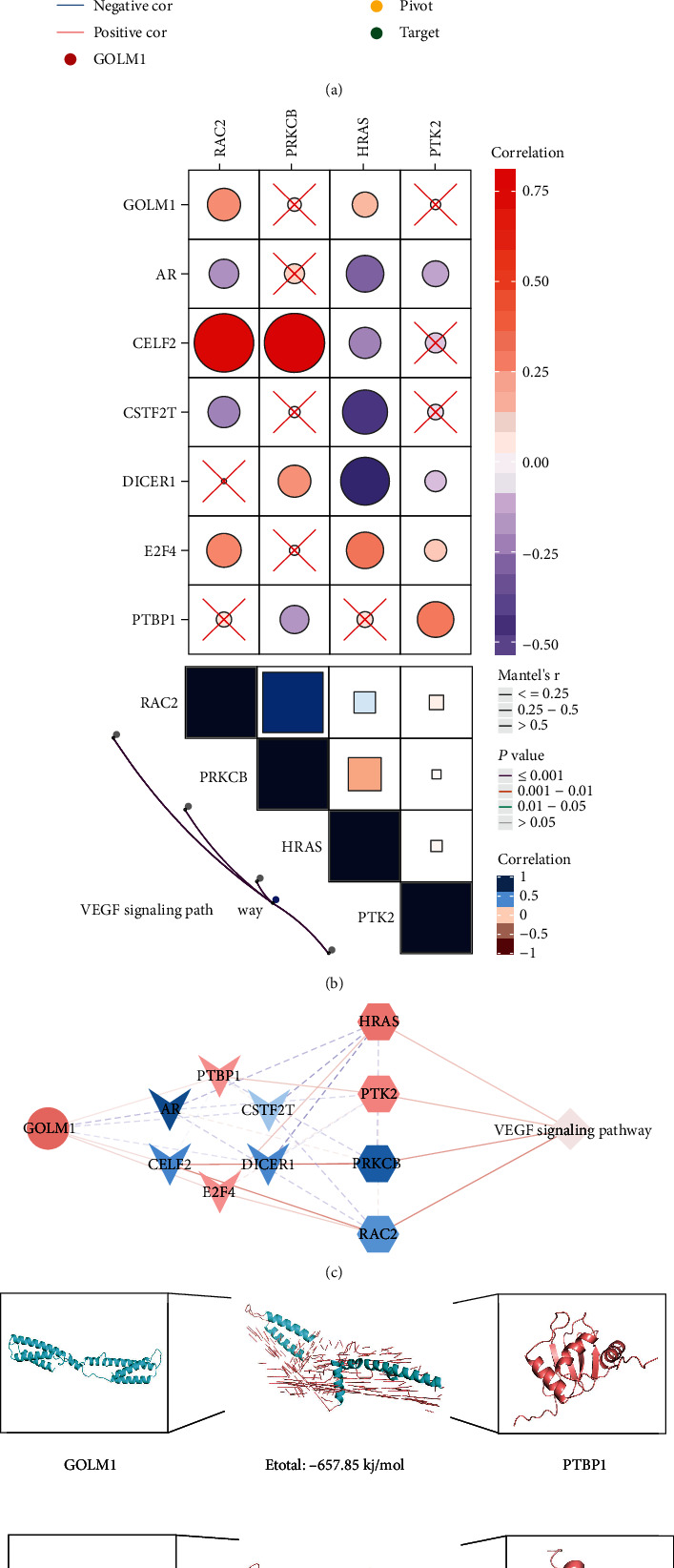
GOLM1 activates the vascular endothelial growth factor (VEGF) signaling pathway to promote angiogenesis in hepatocellular carcinoma. (a) Gene expression correlation network based on GOLM1 as well as pivot and target genes identified in the RNAInter database. (b) Pathway map interrelating GOLM1, pivot genes, pathway genes, and the VEGF signaling pathway. (c) Network view of GOLM1, pivot genes, target genes, and the VEGF pathway. Red represents upregulated gene expression, blue represents downregulated gene expression. (d) Molecular docking studies of PTBP1 and E2F4 with GOLM1. (e) Schematic of a potential mechanism by which GOLM1 promotes angiogenesis in HCC. VEFG: vascular endothelial growth factor.

To identify the regulatory network associated with GOLM1, pivot analysis was performed based on the RNAInter database (http://www.rna-society.org/rnainter) to find the genes interacting with GOLM1. The results identified 37 GOLM1 pivot genes with statistical significance, which indirectly regulated 12 KEGG pathways (Figure 4(a)). In particular, we identified six pivot genes in the VEGF signaling pathway: PTBP1, AR, CELF2, E2F4, DICER1, and CSTF2T. We also identified four pathway genes: HRAS, PTK2, PRKCB, and RAC2 (Figure 4(b)). Thus, a comprehensive regulatory network of GOLM1, pivot genes, pathway genes, and the VEGF signaling pathway was constructed (Figure 4(c)). To further explore the regulation of target genes by GOLM1, we performed molecular docking analyses. The results showed that GOLM1 has the potential to bind E2F4 and PTBP1 (Figure 4(d)). Therefore, we postulate that GOLM1 targets E2F4 and PTBP1 to activate the VEGF signaling pathway, thereby promoting angiogenesis in HCC (Figure 4(e)).

### 3.4. Construction of a Prognostic Model for HCC

**Figure 5:**
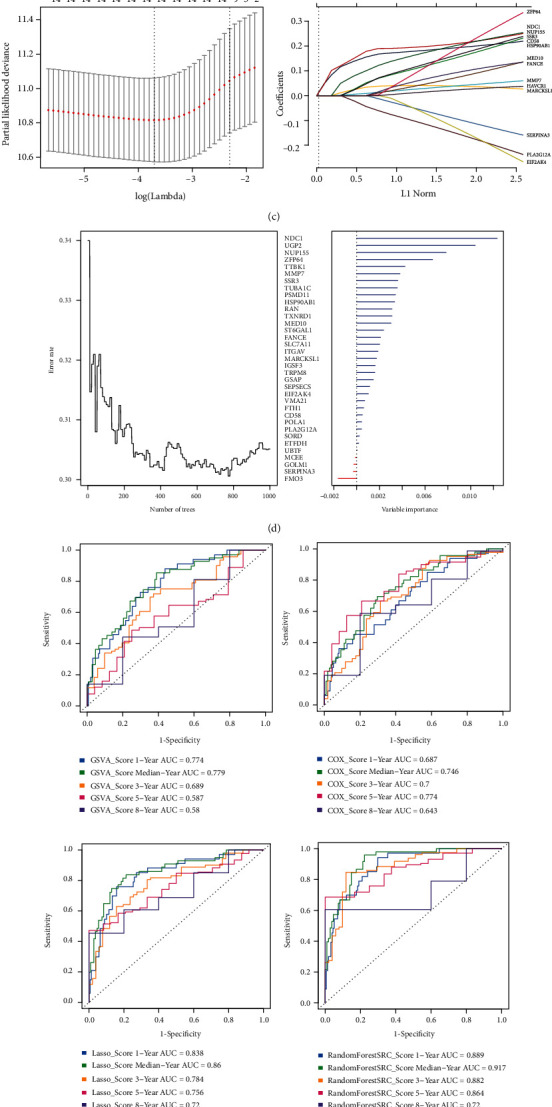
Establishment of the prognostic model for hepatocellular carcinoma (HCC) patients. (a) GSVA score of 53 prognostic genes. (b) Multivariate Cox regression analysis. (c) Establishment of the LASSO regression model. (d) Results of the random forest regression model for selecting prognostic characteristic genes. Thirty-five signature genes were selected by the random forest regression model. (e) The timeROC curve analysis of median survival and survival rates at one, three, five, and eight years for the above four models in TCGA. (f) Nomogram for predicting two- and five-year overall survival rates of HCC patients. The nomogram includes three variables: metastasis, *N* (presence or absence of lymphatic metastasis), and random forest risk score. (g) Performance validation of clinical prognostic models in the TCGA training sets. AUC: area under the receiver operating characteristic curve; GSVA: gene set enrichment analysis; LASSO: least absolute shrinkage and selection operator regression; RandomForestSRC: fast unified random forests for survival, regression, and classification.

In order to screen prognosis-related genes, mechanism genes in Figure 4(c) and 400 specific DEGs were extracted for univariate Cox regression analysis, and 52 DEGs associated with prognosis were obtained. To obtain the best prognostic model, 52 DEGs and GOLM1 were combined to construct four models based on GSVA, multifactor Cox regression, LASSO, or random forest regression. First, the GSVA scores of 53 prognostic genes were obtained by the GSVA model (Figure 5(a)). Then, 53 DEGs were subjected to multivariate Cox regression analysis to screen for independent prognostic factors and construct prognostic models, and finally, three prognostic genes were identified: HAVCR1, ETFDH, and MMP7 (Figure 5(b)). Similarly, LASSO regression analysis was performed on 53 DEGs to further remove redundant variables, and 14 genes were identified and used to construct prognostic models (Figure 5(c)). Finally, we used random forest regression models to identify 35 characteristic genes as the most relevant regulators of prognosis and constructed the corresponding models (Figure 5(d)).

To determine the best prognostic model, temporal ROC curves for median survival and survival at 1, 3, 5, and 8 years were plotted based on the risk scores of the four models (Figure 5(e)). The results showed that the random forest regression model had the optimal scoring efficacy. Subsequently, combined with the clinical characteristics of patients, we incorporated tumor distant metastasis, TNM stage, and random forest score models to construct 2-year and 5-year column line graph prediction models. The results showed that distant tumor metastasis, TNM stage, and random forest score were independent prognostic factors for patients with HCC (Figure 5(f)). To validate the predictive value of the model, survival curves were used to demonstrate the OS and RFS curves of the clinical prognostic score model between samples of the high- and low-risk groups, and the results showed that patients in the high-risk group had significantly lower OS (*P* < 0.0001) and RFS (*P* < 0.0001) than those in the low-risk group, indicating that this clinical prognostic score model could effectively discriminate between the high- and low-risk groups (Figure 5(g)). We confirmed this result in the validation dataset GSE54236 (additional Figures 1(a) and 1(b)).

### 3.5. Role of Protein Processing in the Endoplasmic Reticulum in HCC

**Figure 6:**
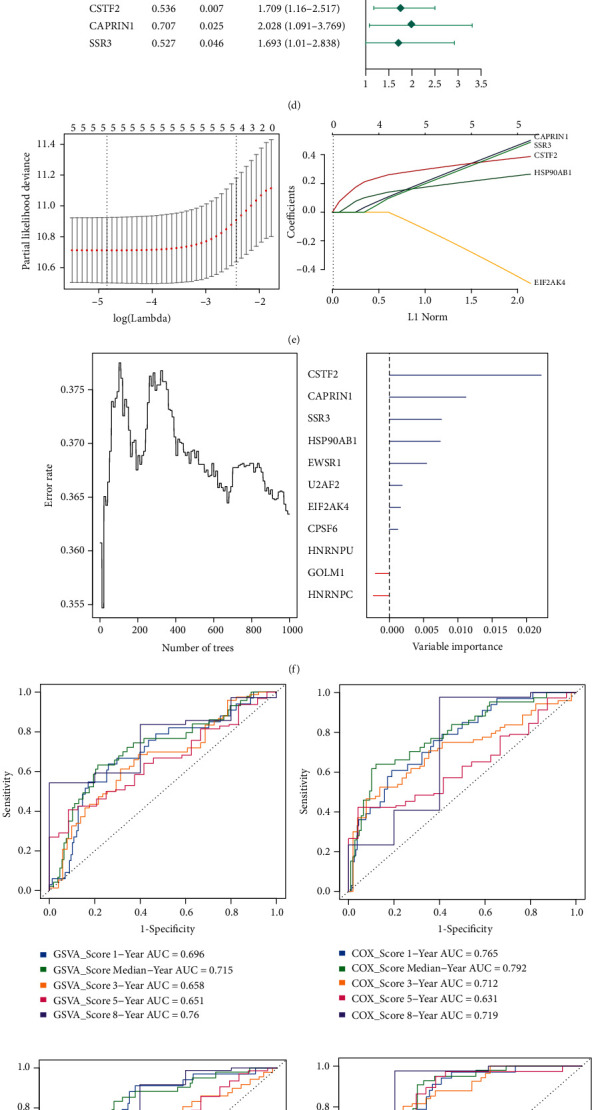
Role of GOLM1 in protein processing in the endoplasmic reticulum in hepatocellular carcinoma (HCC). (a) Regulatory network view of GOLM1, pivot genes, pathway genes, and the protein processing in the endoplasmic reticulum pathway. Red represents upregulated gene expression, and blue represents downregulated gene expression. (b) Molecular docking studies of E2F1 and IGF2BP3 with GOLM1. (c) GSVA score of the 13 prognostic genes. (d) Multivariate Cox regression analysis. (e) Establishment of the LASSO regression model. (f) The random forest regression model identified 11 signature genes associated with survival. (g) The timeROC curve analysis of median survival and survival rates at one, three, five, and eight years for the above four models in TCGA. (h) Performance validation of the optimal random forest regression model in the TCGA training set. AUC: area under the receiver operating characteristic curve; GSVA: gene set enrichment analysis; LASSO: least absolute shrinkage and selection operator regression; RandomForestSRC: fast unified random forests for survival, regression, and classification.

We previously showed that DEGs associated with GOLM1 overexpression are predicted to participate in protein processing in the endoplasmic reticulum (Figure 3(d)) [30]. Multiple pathways, such as the endoplasmic reticulum-associated degradation pathway and the endoplasmic reticulum stress pathway are involved in protein processing in this organelle [30, 31]. Therefore, to explore the potential relevance of protein processing in the endoplasmic reticulum in HCC, we first constructed a comprehensive regulatory network including GOLM1, pivot genes, and pathway genes (Figure 6(a)). We then explored the potential of GOLM1 to bind other molecules in this network. The results predicted that GOLM1 could stably bind E2F1 and IGF2BP3 (Figure 6(b)).

Next, we extracted relevant genes from the comprehensive regulatory network for univariate Cox regression analysis, and 12 genes significantly associated with prognosis were identified. The 12 DEGs plus GOLM1 were combined to construct four models based on GSVA (Figure 6(c)), multifactorial Cox regression (Figure 6(d)), LASSO (Figure 6(e)), and random forest (Figure 6(f)). The random forest regression model with 11 characteristic genes was the best prognostic model (Figure 6(g)). To validate its predictive value, we performed survival analysis according to the random forest risk score: patients with high scores had much worse OS and recurrence-free survival than those with low scores (Figure 6(h)). We confirmed this result in the validation dataset GSE54236 (additional Figures 2(a) and 2(b)).

### 3.6. Immune Infiltration in HCC

**Figure 7:**
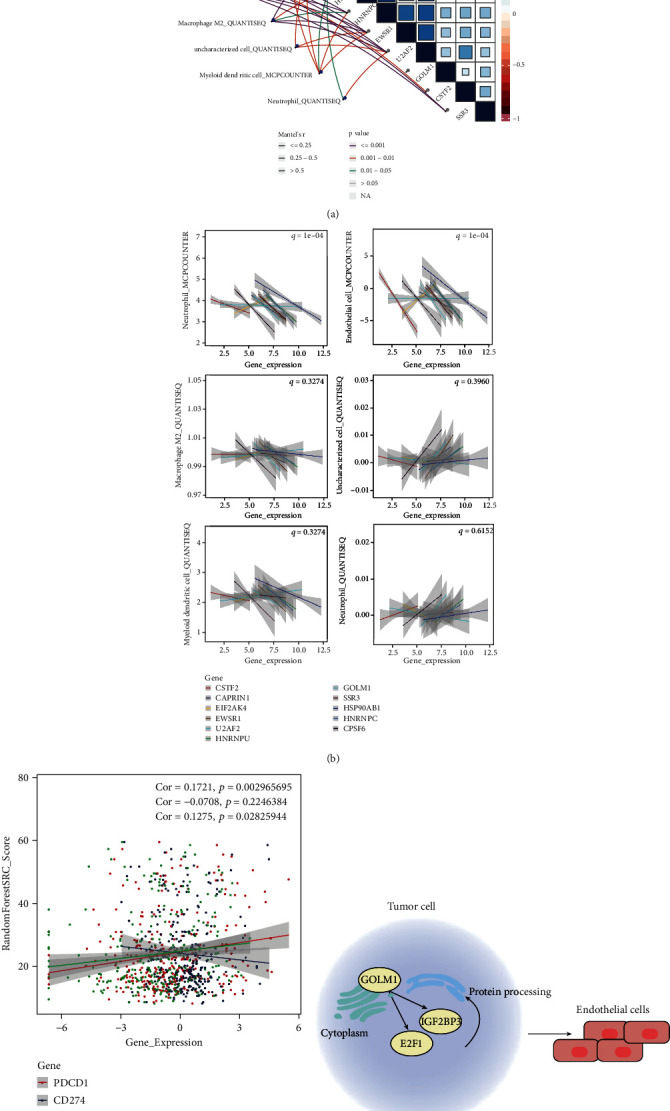
Immune infiltration in hepatocellular carcinoma (HCC) and relationship with GOLM1. (a, b) Correlation between 11 signature genes and immune cells. (c) Correlation between random forest regression model risk scores and immune checkpoints PDCD1, CD274, and CTLA4. (d) Schematic showing a potential mechanism by which GOLM1 may regulate protein processing in the endoplasmic reticulum. GOLM1 binds E2F1 and IGF2BP3 to promote tumor infiltration into the endothelium and affect angiogenesis in HCC.

Studies have pointed out that protein processing in the endoplasmic reticulum plays a crucial role in immune responses [32, 33]. Using the TIMER 2.0 database, we showed that the 11 characteristic genes correlated significantly with the abundance of neutrophils, endothelial cells, M2 macrophages, uncharacterized cells, and myeloid dendritic cells (Figures 7(a) and 7(b)). In addition, we analyzed the correlation between random forest risk score and immune checkpoints PDCD1, CD274, and CTLA4: the risk score correlated positively with expression of PDCD1 and CTLA4, but not with expression of CD274 (Figure 7(c)).

Based on the above results, we propose that GOLM1 may regulate protein processing in the endoplasmic reticulum by binding to E2F1 and IGF2BP3, thereby promoting the infiltration of endothelial cells and angiogenesis in HCC (Figure 7(d)).

## 4. Discussion

HCC is one of the cancers with higher incidence and mortality in the world [34]. Exploring how HCC occurs and progresses may help identify tumor markers, formulate effective treatment plans, and improve prognosis. HCC is a typical vascular tumor, and angiogenesis plays a key role in its growth [4]. However, vascular-related signaling pathways in HCC are still unclear, and new research is urgently needed to find new therapeutic targets. Our study shows that GOLM1 overexpression is closely related to vascular invasion of HCC. Therefore, exploring the relationship between GOLM1 and angiogenesis may help to identify new therapeutic targets in HCC.

First, we used the TCGA database to explore GOLM1 expression in HCC. Consistent with previous studies, we found that GOLM1 was overexpressed in HCC compared with healthy controls and was closely associated with poor prognosis [35].

By analyzing sequencing data in both MHCC97H cells and TCGA database, we identified 400 specific DEGs associated with GOLM1 overexpression. GO analysis revealed that these genes are mainly involved in oxidative stress-related biological processes. KEGG pathway enrichment analysis showed that those genes were involved mainly in key pathways, such as the VEGF signaling pathway and protein processing in the endoplasmic reticulum. In view of the importance of angiogenesis in HCC, it is reasonable to hypothesize that GOLM1 promotes angiogenesis by activating VEGF signaling. The involvement of protein processing in the endoplasmic reticulum is also plausible, because endoplasmic reticulum stress has been implicated in HCC through its ability to promote tumor growth, metastasis, angiogenesis, and drug resistance [36]. In addition, increased oxidative stress is thought to be a recognized mechanism contributing to HCC [37]. Reactive oxygen species (ROS) are a source of oxidative stress generated in various organelles and stress pathways, such as mitochondria, peroxisomes, and endoplasmic reticulum [38]. Excessive ROS disrupts the integrity of proteins and lipids and may cause genetic mutations inducing carcinogenesis [39]. The endoplasmic reticulum generates oxidative stress associated with endoplasmic reticulum stress when it contains endoplasmic reticulum redox protein 1 alpha and protein disulfide bond isomerase [40]. The endoplasmic reticulum oxidative stress triggers the release of hydrogen peroxide and calcium ions into the cytosol, further leading to increased mitochondrial oxidative stress and increased ROS. Hepatic oxidative stress leads to T cell tyrosine phosphatase (TCTPT) inactivation and promotes STAT3 signaling to drive HCC development [41].

Our results showed that GOLM1 might activate the VEGF pathway by binding to E2F4 and PTBP1. E2F4 is a novel tumor marker and well-established transcription factor that has been associated with HCC prognosis [42]. It is involved in the cell cycle, cell proliferation, resistance to apoptosis, and tumor progression [43, 44]. Recent studies reported that E2F4 overexpression is able to promote HCC cell proliferation by upregulating CDCA3 [45]. PTBP1 is an RNA binding protein that regulates RNA splicing and is involved in cellular processes such as the cell cycle, apoptosis, and immune activation [46]. PTBP1 regulates the alternative splicing of exon 10 in the Axl gene, allowing it to promote HCC cell invasion and metastasis [47]. E2F4 and PTBP1 have not previously been linked to HCC angiogenesis, so our results suggest that future studies should explore this possible link in detail.

Our results also suggest that GOLM1 may be involved in protein processing in the endoplasmic reticulum by binding to E2F1 and IGF2BP3, promoting endothelial cell infiltration. Endothelial cells are indispensably linked to angiogenesis, a complex, highly ordered process that is dependent on endothelial cells [48]. Our immunoinfiltration analysis found that endothelial cells were significantly infiltrated; however, the link between GOLM1 and endothelial cells is not known. E2F1 is a transcription factor involved mainly in the regulation of the cell cycle, cell proliferation, and apoptosis [49], and it is a key determinant of the survival of cells under endoplasmic reticulum stress [50]. IGF2BP3 is highly expressed in a variety of tumors including HCC, lung, and prostate cancers, and it helps maintain tumor cell growth, proliferation, invasion, and drug resistance through several oncogenic pathways [51]. IGF2BP3 can inhibit ZO-1 expression, enhancing the ability of HCC cells to invade [52]. Few studies have examined E2F1 and IGF2BP3 in HCC, so our results justify more detailed experiments into how they may contribute to disease onset and progression.

Based on the RNAInter database and VEGF signaling pathway, we identified 11 mechanism genes and then analyzed and verified these genes and 400 specific DEGs in TCGA and GEO databases. Finally, a new HCC prognostic model was constructed based on a random forest approach. Most of the 35 characteristic genes in the model have previously been linked to HCC. For example, low expression of UGP2 is associated with HCC progression [53]; RAN promotes the growth, migration, and invasion of HCC cells [54]; ITGAV is up-regulated in HCC and promotes tumor metastasis [55]; ETFDH is underexpressed in HCC and associated with poor OS [56]; and SERPINA3 mediates the upregulation of HNRNP-K transcriptional activity and promotes the survival and proliferation of HCC cells [57]. Several genes in our model may be related to HCC but the potential connection requires further study. These genes include NDC1, TTBK1, PSMD11, FANCE, TRPM8, GSAP, SEPSECS, VMA21, PLA2G12A, SORD, UBTF, MCEE, and GOLM1.

We provide evidence that the random forest model is an independent prognostic factor for HCC, can be used to predict OS and recurrence-free survival, and can evaluate the prognosis of HCC patients. In order to improve the accuracy of the prognostic prediction, a nomogram was developed based on the random forest model as well as patient clinical characteristics. The OS nomogram also included the risk scores of metastasis, *N* (presence or absence of lymphatic metastasis), and random forest score.

We also identified the optimal random forest regression model based on protein processing in the endoplasmic reticulum. The results of the Kaplan-Meier curve showed that the model can help identify the high-risk and low-risk HCC patients. Among the 11 characteristic genes identified, HNRNPC emerged as an independent prognostic factor for OS and disease-free survival in HCC patients, and it may be related to sorafenib treatment and anti-PD-1 immunotherapy response [58]. A recent study [59] suggested that the splicing regulator hnRNPU is a new transcriptional target of c-Myc in HCC. In that work, c-Myc upregulated hnRNPU, while hnRNPUSSR3 stabilized the c-Myc mRNA, thereby promoting c-Myc-driven HCC development. CPSF6, as an alternative polyadenylation factor, is an activator of pre-mRNA cleavage and polyadenylation processing [60]. CPSF6 is able to upregulate NQO1 to regulate HCC cell metabolism and thereby promote tumor development [61]. EWSR1 is strongly expressed in HCC, it is associated with histological grade and pathological T stage, and it is considered a novel tumor prognostic marker [62]. HSP90AB1 is also associated with HCC, and it may be involved in the progression from cirrhosis to HCC [63]. SSR3 is highly expressed in HCC and is associated with tumor size, TNM stage, differentiation grade, and poor prognosis [64]. CAPRIN1 is upregulated in HCC and can partially reverse the downregulation of c-MYC and CCND2 caused by miR-621 dysregulation, thereby promoting cell proliferation [65]. At present, the roles of EIF2AK4, U2AF2, and CSTF2 in HCC remain unclear. In this way, our results identify novel genes associated with HCC, and future investigation of these genes may provide new insights into the disease and its treatment.

In addition, various types of infiltrating immune cells have been described in the pathogenesis of HCC, and the potential role is not yet clear. To date, the main focus of cancer immunotherapy has been to interrupt immune checkpoints that inhibit antitumor lymphocytes. In addition to lymphocytes, the HCC milieu includes many other immune cell types, of which neutrophils are emerging as important contributors to the pathogenesis of hepatocellular carcinoma. A growing body of evidence supports neutrophils as key mediators of the immunosuppressive environment in which certain cancers develop and as drivers of tumor progression [66]. Little is known about the impact of endothelial cells on tumor cell behavior. In HCC patients, endothelial cells act as promoters of molecular crosstalk, enhancing HCC cell survival, migration, and invasion [67]. Recently, information about M2 macrophages promoting hepatocellular carcinoma metastasis revealed the mechanism of metastasis in HCC [68]. However the role of bone marrow dendritic cells in HCC is not known for the time being.

Although our study identified potential molecular mechanisms through which GOLM1 promotes HCC angiogenesis, it still has several limitations. First, our work was based mainly on bioinformatics predictions using previously published data from TCGA. Nevertheless, we validated our in silico findings using a GEO dataset and explored the role of GOLM1 in HCC using cell culture and RNA sequencing together. Second, the established nomogram model needs external validation. Since our prognostic model was constructed and validated using retrospective analysis of public databases, it should be confirmed in prospective studies. Future work should investigate, in vivo and in vitro, how GOLM1 promotes HCC angiogenesis.

## 5. Conclusions

Our study constructed HCC prognostic models based on DEG associated with GOLM1 overexpression, which may help to stratify HCC patients according to prognosis and to guide individualized treatment. Functional enrichment analysis of these genes led us to propose a mechanism by which GOLM1 promotes HCC angiogenesis. This may help develop effective treatments.

## Data Availability

The datasets analyzed during the current study are available in the TCGA (https://portal.gdc.cancer.gov/) and GEO repository (https://www.ncbi.nlm.nih.gov/geo/).

## Supplementary Materials

Supplementary 1 Figure S1. Validation of the random forest regression model in the GSE54236 validation set. (a) Kaplan-Meier curve of survival probability between the high-risk and low-risk groups in GSE54236. (b) The timeROC curve analysis of the random forest regression model in GSE54236. AUC: area under the receiver operating characteristic curve; OS: overall survival; RFS: recurrence-free survival; RandomForestSRC: fast unified random forests for survival, regression, and classification.

Supplementary 2 Figure S2. Validation of the random forest regression model in the GSE54236 validation set. (a) Kaplan-Meier curves of survival probability between the high-risk and low-risk groups in GSE54236. (b) The timeROC curve analysis of the random forest regression model in GSE54236. AUC: area under the receiver operating characteristic curve; OS: overall survival; RFS: recurrence-free survival; RandomForestSRC: fast unified random forests for survival, regression, and classification.

Supplementary 3 Supplementary Table 1. Details of the 400 specific DEGs associated with GOLM1 overexpression.
