# Supplementary figures and images for: Integrative Analysis Reveals the Potential Role and Prognostic Value of GOLM1 in Hepatocellular Carcinoma
Source: Oxid Med Cell Longev. 2022 Sep 28;2022:8284500. doi: 10.1155/2022/8284500 (PMC9535134; doi:10.1155/2022/8284500)

**A**

## OS Survival Analysis of GSE54236\_Score

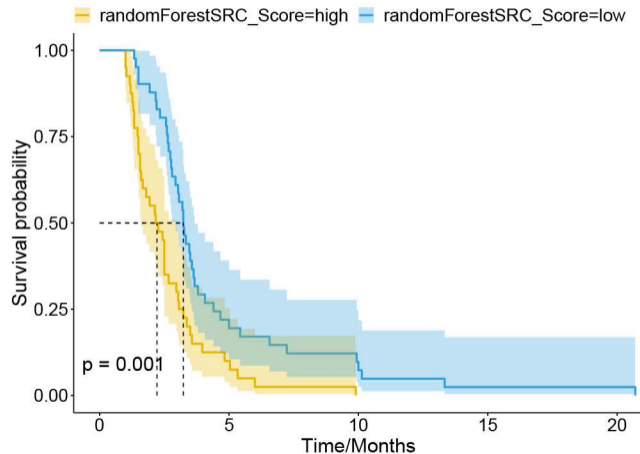**B**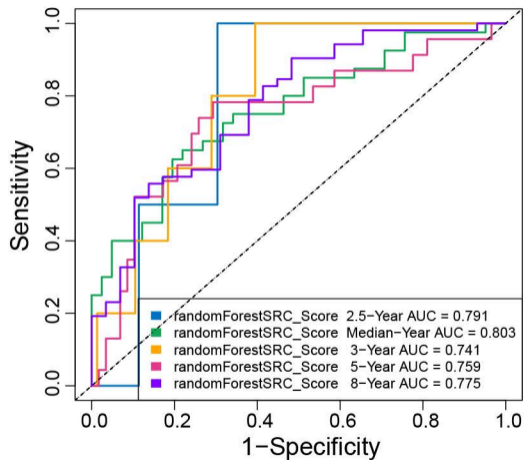

Supplement: Supplementary 1 — Figure S1. Validation of the random forest regression model in the GSE54236 validation set. (a) Kaplan-Meier curve of survival probability between the high-risk and low-risk groups in GSE54236. (b) The timeROC curve analysis of the random forest regression model in GSE54236. AUC: area under the receiver operating characteristic curve; OS: overall survival; RFS: recurrence-free survival; RandomForestSRC: fast unified random forests for survival, regression, and classification. [file 8284500.f1.pdf]

**A**

## OS Survival Analysis of GSE54236\_Score

randomForestSRC\_Score=high randomForestSRC\_Score=low

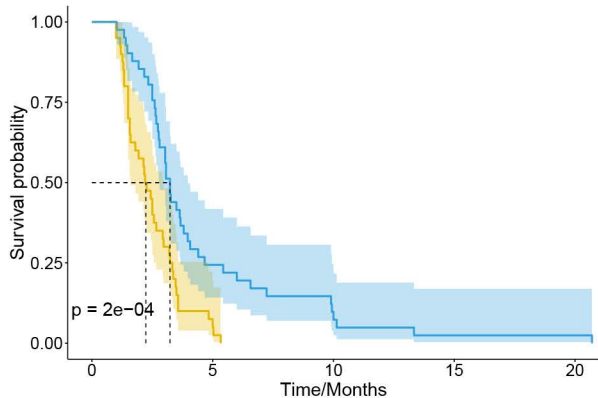**B**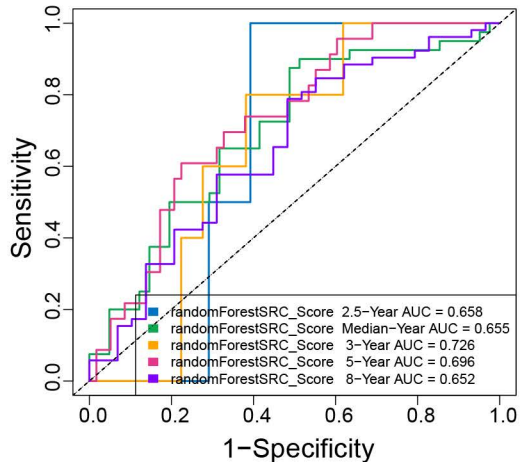

Supplement: Supplementary 2 — Figure S2. Validation of the random forest regression model in the GSE54236 validation set. (a) Kaplan-Meier curves of survival probability between the high-risk and low-risk groups in GSE54236. (b) The timeROC curve analysis of the random forest regression model in GSE54236. AUC: area under the receiver operating characteristic curve; OS: overall survival; RFS: recurrence-free survival; RandomForestSRC: fast unified random forests for survival, regression, and classification. [file 8284500.f2.pdf]
